# Schistosomiais and Soil-Transmitted Helminth Control in Niger: Cost Effectiveness of School Based and Community Distributed Mass Drug Administration

**DOI:** 10.1371/journal.pntd.0001326

**Published:** 2011-10-11

**Authors:** Jacqueline Leslie, Amadou Garba, Elisa Bosque Oliva, Arouna Barkire, Amadou Aboubacar Tinni, Ali Djibo, Idrissa Mounkaila, Alan Fenwick

**Affiliations:** 1 Schistosomiasis Control Initiative, Department of Infectious Disease Epidemiology, Imperial College London, London, United Kingdom; 2 Réseau International Schistosomoses Environnement Aménagements et Lutte (RISEAL-Niger), Niamey, Niger; 3 Department of Epidemiology and Public Health, Swiss Tropical and Public Health Institute, Basel, Switzerland; 4 University of Basel, Basel, Switzerland; 5 Ministère de la Santé Publique, Niamey, Niger; London School of Hygiene & Tropical Medicine, United Kingdom

## Abstract

**Background:**

In 2004 Niger established a large scale schistosomiasis and soil-transmitted helminths control programme targeting children aged 5–14 years and adults. In two years 4.3 million treatments were delivered in 40 districts using school based and community distribution.

**Method and Findings:**

Four districts were surveyed in 2006 to estimate the economic cost per district, per treatment and per schistosomiasis infection averted. The study compares the costs of treatment at start up and in a subsequent year, identifies the allocation of costs by activity, input and organisation, and assesses the cost of treatment. The cost of delivery provided by teachers is compared to cost of delivery by community distributers (CDD).

The total economic cost of the programme including programmatic, national and local government costs and international support in four study districts, over two years, was US$ 456,718; an economic cost/treatment of $0.58. The full economic delivery cost of school based treatment in 2005/06 was $0.76, and for community distribution was $0.46. Including only the programme costs the figures are $0.47 and $0.41 respectively. Differences at sub-district are more marked. This is partly explained by the fact that a CDD treats 5.8 people for every one treated in school.

The range in cost effectiveness for both direct and direct and indirect treatments is quantified and the need to develop and refine such estimates is emphasised.

**Conclusions:**

The relative cost effectiveness of school and community delivery differs by country according to the composition of the population treated, the numbers targeted and treated at school and in the community, the cost and frequency of training teachers and CDDs. Options analysis of technical and implementation alternatives including a financial analysis should form part of the programme design process.

## Author Summary

Schistosomiasis and soil-transmitted helminth control programmes are important, relatively low cost means to improve the health of those affected, in particular rural school age children. It can also reduce schistosomiasis related morbidity in their later lives. The paper presents information on the implementation and costs of a large scale national programme in Niger. The total economic cost per treatment was $0.58. This includes programme, government and international costs. Two systems, school based and community delivery were used to treat children and targeted adults. Contrary to findings in some countries we find that school based delivery is less cost effective than community delivery. This is due to the low proportion of the population targeted and treated by the school based system. Treating adults as well as children increased the numbers treated and reduced the overall cost per treatment. Prevalence and infection is higher in children than adults and overall effectiveness in terms of infection averted is affected. The cost per infection averted is assessed for direct treatment and direct and indirect treatment effects. The study expands the evidence available for decision makers involved in programme planning and design, funding and implementation.

## Introduction

Schistosomiasis is one of the most prevalent chronic infectious diseases found world-wide and is associated with anaemia, chronic pain, diarrhoea, and under nutrition. It is recognised as a major public health problem in many rural areas, particularly in school-age children. With affordable and sustained control measures morbidity and transmission can be decreased.

Robust studies on the implementation of large scale control of schistosomiasis and soil transmitted helminths (STH) are required to strengthen the evidence base on the cost-effectiveness and affordability of such investment [1]–[3]. In particular, evidence on effectiveness is needed to support the strategic planning for expanded treatment and global coverage as well as for national vertical and integrated Neglected Tropical Disease (NTD) programmes. The objectives of this study are to identify: the cost of the Mass Drug Administration (MDA) programme; the cost per person treated; and the costs of treatment as delivered by school based staff and community distributers.

A number of studies have identified the costs of targeted and MDA for schistosomiasis control [4]–[9]. These have, with the exception of [4], [9], provided empirical evidence of school based approaches. This paper provides the cost of MDA treatment and compares the costs of the school and community based distribution systems used. It assesses the evidence from other MDA programmes taking account of factors such as the level of school enrolment, coverage levels to consider the general guidelines that can be taken from these works.

### Description of the Programme for Mass Drug Administration in Niger

In 2004 Niger established a national programme to control schistosomiasis and soil-transmitted helminths (PNLBG) supported by the Schistosomiasis Control Initiative (SCI), funded by the Bill and Melinda Gates foundation [10]. Its objective in line with Resolution WHA54.19 was to treat 75% of school age children at risk of infection and in communities where prevalence is over 50% to also treat at risk adults. The purpose being to reduce the morbidity related to schistosomiasis infection to a level at which it would not constitute a public health problem [11].

The primary school net enrolment rate (NER) in 2004 in Niger was 41% (UNESCO UIS global education database Table 5, Enrolment ratios by International Standard Classification of Education (ISCED) level), lower in rural areas; and considerably less than the rate of 68% for Sub Saharan Africa (SSA). To achieve high treatment coverage in targeted school age children and at risk adults two treatment strategies, school-based and community-based distribution, were established. Treatment for *S. haematobium* was provided every two years in most endemic areas, and annually in high prevalence areas to reduce initial levels. School-based distribution was provided by trained teachers who distributed the drugs to students in the schools. Children not attending school could receive treatment either in the schools or from the Community Drug Distributor (CDD) at home or at another fixed treatment location.

**Figure 1:**
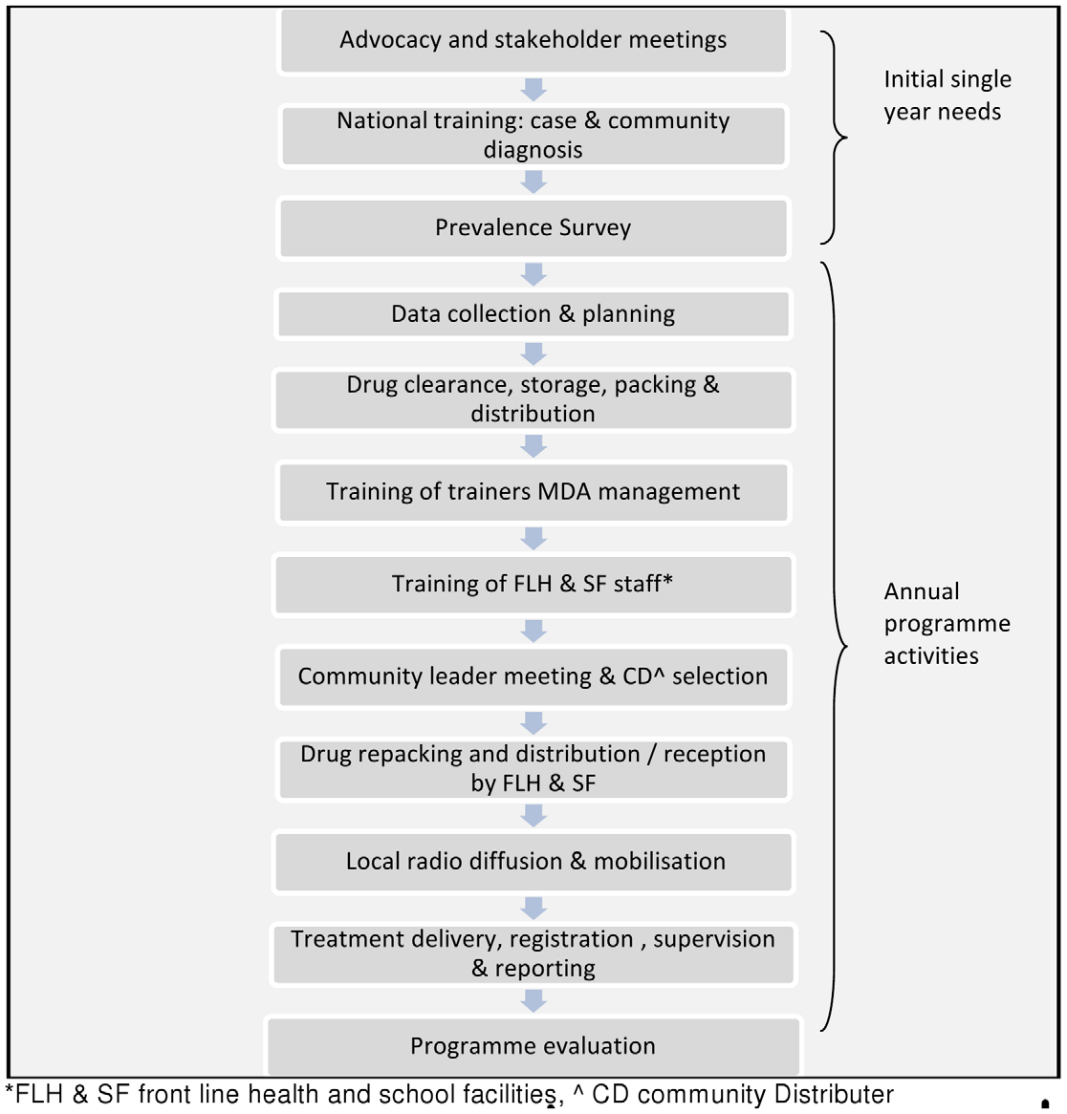
The main steps in the MDA programme. Figure 1 presents the component activities of the MDA process. Activities mainly occurring in the first year are shown as well as annual activities.

The MDA programme was established and rolled out over 2 years from April 2004 across 40 districts in all 7 regions of the country, including the capital city Niamey. The programme activities were implemented progressively commencing in the Tilaberi and Dosso regions. In 2004/05, 1,627,828 treatments were delivered in 22 districts and in 2005/06 2,683,121 treatments were delivered in 40 districts. Figure 1 outlines the main MDA programme activities.

Initial meetings and agreement of the programme with the regional and district administrations were followed by a prevalence survey and mapping to prioritise areas for MDA. A national workshop and practical field sessions to develop capacity in the diagnosis of schistosomiasis was organised. Further capacity-building workshops and training for staff in organisation, management and implementation of MDA was provided to key district health and educational inspectorate staff in an initial national workshop. These staff then trained clinic staff, health workers and head teachers though district meetings. Training was provided on the calculation of drug requirements, drug distribution organisation, the management of side effects and the reporting of results. Community and school-based drug distributors were trained in the use of dose poles (to determine drug dosage), and the completion of treatment forms.

The national programme developed, piloted, printed and delivered information, education and communication (IEC) materials for distribution to the districts. These materials included posters and a booklet for use in schools and communities as well as technical sheets for those administering the drugs. Drugs were procured centrally by SCI on behalf of countries which SCI supported in West and East Africa [12]. The drugs were sent directly from the National store to the districts. The districts and inspectorates repacked the drugs and IEC material for distribution to or collection by clinics and schools. Social mobilisation activities were undertaken at various administrative levels. National radio and television broadcasts were undertaken in three local languages and in French, organised by the PNLBG; local radio broadcasts were organised by the districts; village criers were organised by clinics to inform communities about the logistics of the MDA.

A rally to launch the campaign was undertaken and organised by an host region; it involved a day of speeches, dance and hospitality supported by national, regional and programme dignitaries and was broadcast on national radio and television.

Treatments were delivered at schools by teachers; CDDs provided treatment by going from door to door and at other fixed points supervised by clinic, district and regional staff. Technical and management support and supervision were provided to the districts by national staff.

At the end of the MDA unused drugs and monitoring reports were collected by national staff. A one day post MDA evaluation meeting was held in each participating region attended by national, regional and district staff. A summary of the partner roles and responsibilities is identified in Table 1.

**Table 1:** Roles and responsibilities in the Niger schistosomiasis and STH MDA.

National	Region & Department	Clinic, School, Community
• Advocacy meetings with national, regional and district health and education administrations	**Regional**	• Training by clinic nurse of community distributers
• Organisation of national prevalence survey	• Disbursement of funds to the districts, and	• The school head undertakes the training of school staff for school based MDA.
• Training for diagnosis	• Supervision of district MDA activities	• Engagement of village criers to publicise community MDA
• Drug clearance and reception	**District**	• Organisation of MDA drug delivery to the communities and MDA supervision of distributers
• Storage, repacking and delivery of drugs and materials to districts	• Training of primary school heads and clinic health staff;	• Supervision by Clinic Head Nurse of drug delivery by CDD and in schools by headmasters
• Central training of trainers for regional and district health and education staff	• Repacking and delivery of drugs and materials to the clinics and to the sector education authorities or schools	• Collation and reporting of treatments by clinic nurse and by head teacher to inspectorate
• Support for national campaign inaugural rally	• Supervision by district and inspectorate of drug delivery in communities and schools.	• Response to secondary effects reported to clinics initially
• Supervision, technical support of district MDA organisation & delivery	• District level radio emission and diffusion	• Disbursement of moneys for community MDA
• Collection of surplus drug supplies and coverage forms	• Disbursement of moneys to the clinics	
• Programme evaluation		
• National radio and television diffusion		

## Methods

A protocol for the cost and resource use study was agreed with Niger Ministries of Health and Education at national and local government level. Written informed consent to participate in the programme longitudinal surveillance and monitoring research was obtained from the children's parents or guardians, or head teachers according to the study protocol approved by St Mary Research Ethics Committee of Imperial College, UK, 2003, (EC No 03.36, R&D No: 03/SB/003E) and amended 2005 St Mary's (REC Ref: AM2003).

This was a retrospective study which covered a two year period from April 2004 to May 2006, including the first and second years of MDA and related programme activities in four health districts.

All data on first year costs at national, regional, district, and sub district levels were taken from the PNLBG accounts and receipts and records of staff missions or activities. Second year cost data for national and regional level activities were taken from receipts. District and sub district, school and community MDA resource use data for 2005 were collected in June 2006 through a retrospective survey. The four health districts: Kollo, Tera, Tilaberi, (Tilaberi region) and Gaya (Dosso region) are all located in the Niger River Valley, and were in the first phase of implementation. The control programme had previously established sites for longitudinal monitoring of prevalence and morbidity in these districts.

The cost survey was designed to collect data on the time taken and resources used by district and sub district health staff, and by the education inspectorate and school staff in the 2005 MDA delivery. Questionnaires were designed and tested, and a 2 day training workshop was undertaken in 2006 to familiarise and train the schistosomiasis MDA district co-ordinators, responsible for the data collection.

The questionnaires covered the usage of: vehicles, fuel and other equipment and materials used in the MDA and in training, time spent in different activities by staff at regional, district, and clinic level, payments made to CDDs, and for local services. Survey data on 2005 MDA delivery costs were verified with PNLBG receipts. Drug usage was obtained from district and from PNLBG records. Coverage figures were obtained from district treatment registers and the national annual treatment summary.

Longitudinal surveillance and monitoring data were obtained from records of the Centre de Recherche Medicalé et Sanitaire (CERMES) and from the PNLBG register of activities and receipts.

### Determination of the economic cost of treatment

The study examines the economic costs of the MDA programme in its first and second years. The economic costs include the full value of the resources used. Where this is not adequately represented by the financial or market cost, an opportunity cost is used (see Table S4). The main cost elements include: the programme specific expenditure; the opportunity cost or value of government contributions related to in-kind costs of using local government staff and vehicles and the value of CDD's time (taken as the daily agricultural labour rate); and the international costs of programme co-ordination, reporting and technical support.

Programme costs include directly incurred capital costs; recurrent costs; and variable costs. Capital costs incurred by the programme included central level purchase of Information Technology (IT), medical and laboratory equipment and other electrical and mechanical goods and furniture used to equip the PNLBG office including the purchase of four 4×4 vehicles for PNLBG (Table S1). Capital costs were annualised over their useful lives (Table S2) using a discount rate of 3%. This represents the annual cost of owning and operating an asset over its lifespan.

Programme recurrent costs including staff costs, office and vehicle running costs, and programme variable costs were collected from the programme records, accounts and receipts. These costs were apportioned in relation to the time spent by programme staff on MDA activities and the proportion of that time allocated to study areas.

Variable costs related to perdiems, materials and services incurred in relation to the programme activities. Centralised activities (e.g. organisation and provision of national training for all district technicians, planning and organisation) and regional activities (e.g. MDA launch) were equally apportioned in relation to the number of districts in the MDA and share of the four study districts. Location specific activity costs such as supervision, mapping, central delivery of drugs to districts were allocated on the basis of costs incurred in the study districts.

Sentinel monitoring informs the national treatment strategy. The costs of sentinel site monitoring were apportioned to the study districts on the basis of study area treatments relative to national treatments.

Government staff costs were based on salary costs collected through questionnaire and the Government salary grid. District and sub district vehicle usage was calculated from questionnaire returns and costed using hire rates. These values are estimated to reflect the opportunity cost of using the resources for the MDA rather than for an alternative activity.

Costs were collated and classified by three levels of organisation (national, regional & district, or community), type of activity (training, support & supervision, baseline & monitoring, reporting, evaluation, advocacy, mobilisation & IEC) and cost type (fuel, transport, materials, services, drugs, per diems, temporary contracts and office related recurrent costs).

Prices are in constant 2005 terms (Table S4). Foreign exchange was converted at the fixed rate of CFA 655/Euro and $1.244/Euro (http://www.federalreserve.gov/release/January 2 2009). Discounted economic analysis was undertaken using discount rate of 3% in line with World Bank rates [13]. The cost of a treatment includes both albendazole and praziquantel.

### School based and community delivery system cost calculation

Community and school based delivery was and is practised nationally. The costs incurred by the two systems were equally attributed at national, regional and district level. It is at sub district level that the systems differ in the organisation and implementation of the delivery activities. The school and sub district delivery services used a partial analysis which took account of these cost components only.

The cost of delivery using a CDD and of using a teacher was calculated. These costs included per diems and travel allowances for CDD and head teacher training; allowances for delivery (applicable only for CDD), health clinic staff costs for CDD selection (per diems and fuel) and supervision (fuel only). The training of one or more teachers and their supervision in schools was undertaken by the school head, no financial cost was incurred. Joint activity costs of the district health and education inspectorate (training, drug repacking, drug delivery to sub districts and schools and supervision) would be incurred despite the system. These have not been included in the partial analysis but an allowance has been estimated to allow comparability with other MDA programmes.

### Cost effectiveness analysis

The effectiveness of treatment was calculated as the difference between the population with schistosomiasis infection at baseline and follow-up survey. The prevalence rates used are from a longitudinal health impact study (Nadine Seward (2007) Niger Three Years Data Analysis, SCI internal report (unpublished)).

To assess the effectiveness of the programme's direct and direct and indirect treatment effects an assessment of the impact in the treated population and in the targeted population was made. Treatment costs were calculated as the number of treatments in each year multiplied by the full economic cost in 2004/5 and in 2005/6.

Eight schools and four communities located in areas highly endemic for schistosomiasis took part in a longitudinal health impact study. The study used baseline and longitudinal follow-up surveys one year post treatment to monitor: parasitological indicators (prevalence and intensity of helminth disease examining stool and urine samples following standard procedures using kato katz and filtration methods [14]); morbidity indicators (anaemia and associated pathology of schistosomiasis, assessed by ultrasound examination following standard protocols developed by WHO) and general indicators of height and weight. The baseline survey enrolled 1659 children from 8 different schools in 3 regions prior to the first MDA campaigns of 2004 and 2005. The number of children enrolled from each school ranged from 179 to 299; with almost equal numbers of children in age groups of 7, 8 & 11 years old. Of those recruited 1193 (72%) were followed-up successfully at year 1 and year 2 surveys. Adults and adolescents were monitored in 4 sites in a single region. A total of 484 adolescents and adults were recruited at baseline. Of these, 143 (30%) were followed-up successfully at both year 1 and year 2 surveys. The sample sizes was estimated using the same criteria as described in [15].

The surveyed sites mirror the MDA treatment and represent MDA performance in targeted populations taking into account the treated and untreated participants in proportion to the MDA coverage. Any indirect effect of reduced infection in untreated pupils resulting from changes in the force of infection is reflected in the intensity of infection [16] which is related to prevalence ([17] provides more detail). To assess the wider impacts on the community, untreated first year students were monitored in the schools. Adults and adolescents were monitored at four sites.

## Results

### Total economic costs of treatment

The total economic cost of the programme including programme specific expenditure, national and local government costs and international technical support and programme co-ordination in four study districts, over two years, was US$ 456,718 (Table 2); an economic cost per treatment of $0.58. Excluding international costs, the programme and government expenditure was $0.54 per treatment. The programme expenditure per treatment was $0.44. The average drugs cost was $0.28 per treatment. The numbers treated in these two years totalled 818,562 (781,883, discounted at 3%).

**Table 2:** Discounted economic cost of the MDA programme for April 2004 to March 2006 in 4 districts (2005 prices).

		Regional	School, clinic	International		Cost
Cost Category	National	/District	& community	TC & drugs	Total	Distribution
Programme expenditure						
Capital	10,226				10,226	2%
Recurrent	21,154				21,154	5%
Variable	35,532	17,667	35,261		88,460	19%
Drug cost				222,385	222,385	49%
Total Programme	66,912	17,667	35,261	222,385	342,226	
Programme cost	66,912	17,667	35,261	222,385	342,226	75%
Government cost	3,585	10,721	67,559		81,865	18%
International tech. support				32,627	32,627	7%
Total Economic Cost	70,497	28,388	102,820	255,013	456,718	100%

The distribution of costs between the programme, the government and international support are shown in Table 2. Drugs accounted for 49% of the total economic cost (65% of programme expenditure), variable costs accounted for 19% of the economic cost (26% of programme expenditure). Overall there was little difference in the total economic cost of the programme in the four districts between the first and second years. However the total economic cost per treatment in the first year was $0.68 and in the second year was $0.51. Cost differences are shown in Table 3 and discussed below.

**Table 3:** Annual economic cost of the MDA programme in four districts (2005 prices).

Costs	2004/05	2005/06	% change
Programme cost	75,421	49,455	−34%
Government cost	43,646	41,894	−4%
International tech. support	24,082	9,810	−59%
Drug cost	103,653	129,166	25%
Total costs	246,802	230,326	−7%
Total costs excl. drugs	143,149	101,159	−29%
Total costs discounted (3%)	239,614	217,104	
Number treated	364,593	453,969	25%

Note costs are not discounted.

**Figure 2:**
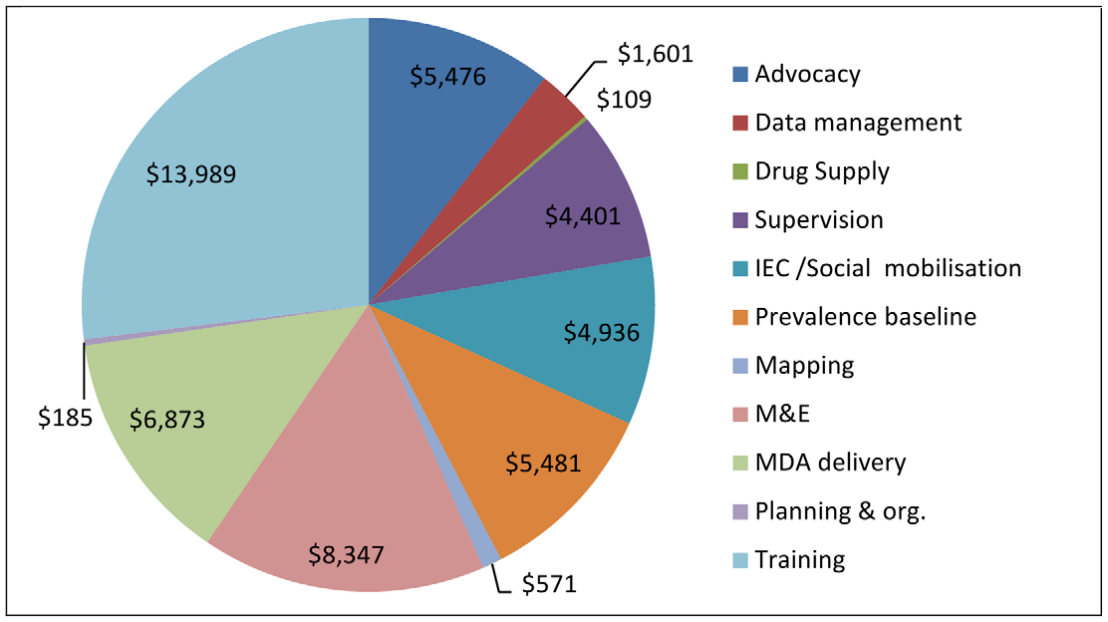
2004/05 Variable costs by activity in 4 districts (2005 prices). Total variable cost was $51,970 in the 4 districts. This includes start up costs involving advocacy, the prevalence baseline, development of IEC materials and establishing monitoring sites. At sub district level, planning and organisation is undertaken at the same time as training.

**Figure 3:**
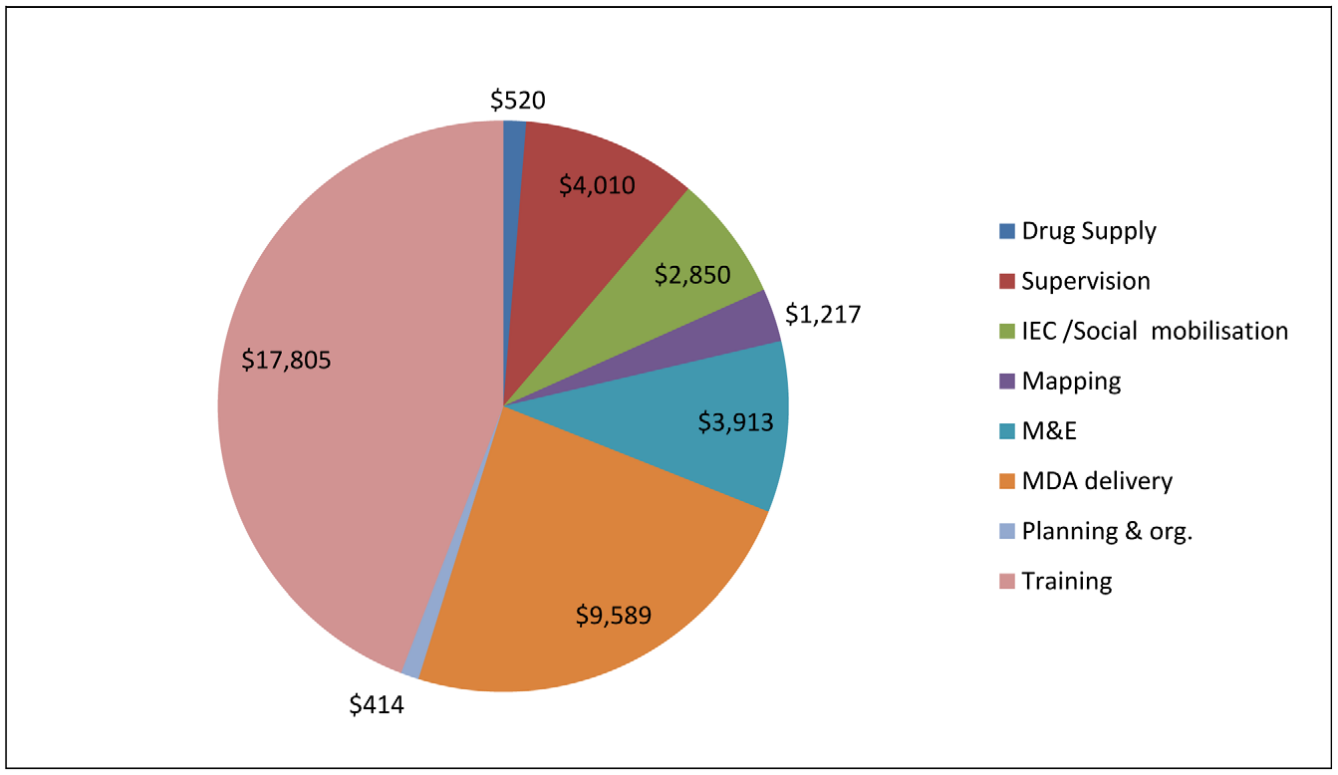
2005/06 Variable costs by activity in 4 districts (2005 prices). Total variable cost was $40,318 in the 4 districts. At sub district level, planning and organisation is undertaken at the same time as training. Compared with the previous year, 25% more people were treated.

Excluding the MDA drug costs, the economic cost of the programme in the four districts in the second year was 29% less costly than the first year and treated 25% more people. Higher costs in the first year of the programme are seen in programme costs and international support. Three factors contribute to this. The cost of the initial start up activities incurred in the first year only. The activities involved advocacy, development of IEC materials, prevalence surveys and data collection for planning and the establishment of monitoring and evaluation (M&E) activities, in particular the longitudinal monitoring sites (illustrated in figures 2 and 3), and repair and maintenance of the national office. In the second year the programme was scaled up. This reduced the apportioned share of recurrent and capital programme costs and international costs allocated to the study area. In 2004/05 22 districts were treated and in 2005/06 40 districts were treated. Within the study area the population treated in the second year which was 25% more than those treated in the first year.

The distribution of variable expenditure (excluding drugs) by activity in the study area is presented in Figures 2 and 3. These show the relatively large proportion of expenditure on training and on MDA delivery. It also highlights activities mainly undertaken at establishment. Total programme variable costs in 2004/05 were $ 51,970 and in 2005/06 were $ 40,318, 22% less than those in the first year.

M&E costs include costs of process monitoring in 2004/5, annual district and regional evaluations and programme health impact monitoring undertaken through the National sentinel sites. These costs amounted to an average of 13% of variable costs over the 2 years. Table 4 presents the average allocation of cost by category (capital, recurrent and variable) and type of input. Labour related costs (salary plus per diems) and vehicle and fuel costs account for 64% and 19% of all costs excluding drugs.

**Table 4:** Programme and government MDA costs (2004/06) allocated by cost category.

Costs	% distribution*
Capital Costs	5%
Recurrent Costs	
Salary	38%
Vehicle & office fuel	10%
Office & other	2%
Communications	1%
Variable Costs	
Perdiems	26%
Transport	4%
Fuel	5%
Material & services	9%
Total	100%

*Percentage based on discounted cost for the 2 years, $201,705 excluding drugs and international costs.

Sensitivity analysis was undertaken on major cost items. A 10% increase in the cost of drugs would result in a 4.9% increase in the total economic cost of treatment ($456,718), and a 6.5% increase in the current programme cost ($342,226). A 10% increase in perdiems and allowances would result in a 1.1% increase in the total economic cost of treatment, or a 1.5% increase in the programme cost, a 4.2% increase in the programme cost excluding drug costs. A 10% increase in wages and salaries would result in a 1.5% increase in the economic cost of treatment. It would impact most on the government sector and distributer opportunity costs increasing costs by 7.7%. The increase on the programme cost would be 0.2% The sensitivity of total economic cost to a saving in teacher training costs was explored. This assumed community distributers would undertake the school treatments for the same fixed allowance. Savings in teacher training allowances are assumed, but not the economic cost of their time which would still be required to support distributers in school based treatment. Any savings in teacher time would be offset by the increased opportunity cost of time for community distributers. The impact of the net saving on the total economic cost would be 2.9%. This is equivalent to a saving of 4.1% in the programme cost. This provides an approximate scale of magnitude within which to assess comparative costs of sub district delivery systems below.

### Cost of community based and school based delivery systems

Sub district costs (i.e. clinic, school and community costs) account for the largest portion of the economic cost by administrative level. This is 23% of the total economic cost (based on Table 2); so, it is important to understand the allocation and usage.

Sub district variable programme costs include head teacher and CDD per diems for training and CDD payments for distribution. Sub district government costs include the opportunity cost for the use of motorbikes (11%) and labour (89%). The opportunity cost of labour is principally accounted for by the time of the teacher and head teachers (61%), of the clinic staff in supervision (20%) and CDD time for training and distribution (19%).

Table 5 presents the characteristics and costs of sub district delivery. The economic cost per school based treatment and per CDD treatment delivered was $0.36 (range $0.26–$0.55) and $0.06 (range $0.04–$0.07) respectively. The programme cost per school based treatment and per CDD treatment delivered was $0.09 (range $0.07–$0.15) and $0.03 (range from $0.03–0.04) respectively.

**Table 5:** Characteristics and cost of community and school based delivery in 4 districts 2005/06.

District	GAYA	KOLLO	TERA	TILLABERI
*Characteristics*				
No. senior clinic nurses (1 per clinic)	17	16	19	18
No. community drug distributers (CDD)	206	156	300	291
No. schools in campaign	267	324	275	198
Teacher/CDD ratio	1.30	2.08	0.92	0.68
*Population Targeted & Treated*				
Targeted village population*	103,064	78,084	150,108	145,290
Targeted school related population*	26,872	35,207	32,767	23,266
Treatments by CDDs	75,982	55,820	152,710	103,825
Treatments by teachers	25,121	19,715	12,406	17,245
Treatments administered/CDD	369	358	509	357
Treatments administered/teacher	94	61	45	87
Coverage in villages %	74%	71%	102%	71%
Coverage in schools %	93%	56%	38%	74%
Treated adults	46,653	37,402	69,801	58,895
Treated children	54,450	38,133	95,315	53,320
Overall coverage	78%	67%	90%	72%
*Sub District Financial Costs of Treatment*				
Teacher cost/treatment $	0.07	0.11	0.15	0.08
CD cost/treatment $	0.04	0.04	0.03	0.04
*Sub District Economic Costs of Treatment*				
Teacher cost/treatment $	0.26	0.41	0.55	0.28
CD cost/treatment $	0.07	0.07	0.05	0.06

*Targeted populations at school and in the village include both adults and children.

Source: 2005 Survey data, unpublished programme planning data & treatment data 2005/6 campaign.

The full economic delivery cost of school based treatment in 2005/06 was $0.76, and community treatment was $0.46. If only programme costs are included this figures are $0.47 and $0.41 respectively.

The difference in costs is in part explained by the fact that a CDD delivers 5.8 treatments for every one delivered in school. On average each CDD delivered 407 treatments while each school delivered 70.

### Cost effectiveness of treatment

Over the 2 treatment cycles 530,300 treatments were provided to an estimated 317,549 adults and 288,262 treatments were provided to 241,218 children in the study areas in the regions of Dosso and Tilaberi. Coverage in the target population in Gaya, Dosso was 78% in both years, and was 69% and 71% in the three districts monitored in Tilaberi.

Two estimates of the cost of treatment per case of infection averted (Table 6) are presented. One includes only the direct impacts of treatment and the other includes the direct and indirect impacts of treatment. They provide minimum and maximum limits of the true value. This is discussed further in the next section.

**Table 6:** Cost per infection of schistosomiasis averted for children and adults in four districts of Niger.

Region/Age	No. Treatments	No. People Treated	No. Targeted	Base Prevalence*	Follow up Prevalence*	Infection Averted (Targeted)	Infection Averted (Treated)	Treatment cost $**	$/Infection Averted (Treated)	$/Infection Averted in (Targeted)	% Difference
Tilaberi –children	227,268	186,768	270,678	93.33%	33.57%	111,613	161,757	122,792	1.10	0.76	45%
Dosso –children	60,994	54,450	69,808	72.37%	19.30%	28,897	37,047	32,219	1.11	0.87	28%
Study area: All children	288,262	241,218	340,486	140,509	198,804	155,011	1.10	0.78	42%		
Adults 2004∧	317,549	317,549	446,180	34.12%	18.43%	49,823	70,006	215,933	4.33	3.08	41%
Adults 2004 & 2005∧	530,300	317,549	446,180	34.12%	18.43%	49,823	70,006	324,436	6.51	4.63	41%

*Prevalence rates, SCI internal reports (unpublished). Base and follow up figures are significantly different at 95%CI (Table S3).

**Based on full economic costs of $0.68 (2004) and $0.51 (2005)/treatment.

**∧:** Prevalence relates to baseline and follow up sample for year 1 in Tahoua region, no adults were monitored in the study area. Drop-out rate at follow up year 2 were high and sample composition differed from baseline.

Including only the direct impacts on the treated population the average cost per infection averted in treated children in the 4 districts over the two years was $1.10. Of the 317,549 adults treated in a single round the cost per infection averted was $4.4 and over two rounds it is estimated to be $6.5. The overall cost per infection averted in the treated population of children and adults is calculated as $2.5.

If indirect treatment effects are included the average cost per infection averted in targeted (treated and untreated) children in the 4 districts over the two years was $0.78. Of the 446,180 adults targeted in a single round the cost per infection averted was $3.08 and over two rounds it is estimated to be $4.6. The overall cost per infection averted in the targeted population of children and adults is calculated as $1.78.

The higher cost of infection averted in adults reflects the lower base prevalence rate. The longitudinal adult cohort followed up over the 2 year period suffered high drop-out rates and its composition was significant different at the 0.05 significance level. Males in particular those who were infected with *S. haematobium* infection were more difficult to retain at follow-up. The resulting cohort of 116 adults had a lower proportion of males, and had a lower base rate of infection (24.1%, (95% CI:16.35–31.93)) as compared with the original baseline sample (39.62% (95%CI:35.23–44.01)). To avoid this issue the results for the sample monitored at the first year follow up are used and it is conservatively assumed that the prevalence in the second follow up did not change.

## Discussion

The cost per treatment and prevalence figures relate to the study sample of four districts located in the Niger River Valley. This was and is an area of high disease prevalence and high population density relative to other parts of the country. The costs per person treated may be higher in lower density and more remote areas. Likewise the cost per infection averted will be greater in sub populations with lower changes in prevalence.

The cost study relied on the survey work for details of district and sub district resource use. As the survey was undertaken almost a year after the MDA, it may have been affected by recall bias. A further limitation of the study is the delay in final analysis. This limited follow-up work; the recent MDA's confuse recall and some key people have changed positions and locations. However, the issues raised by the study analysis are still relevant and worthy of further investigation.

One of the strengths of the study is the availability and use of MDA health impact monitoring results to assess programme effectiveness. Cost effectiveness studies are often obliged to use trials data concerned with the efficacy of treatment [3] rather than programme effectiveness as monitored in Niger. This potentially allows us to capture direct and indirect treatment effects as identified by Miguel and Kremer [18] and French et al [16]. Both identify direct and indirect effects of treatment in terms of the reduced transmission of *Schistosoma mansoni* in children in the community including children not treated. The direct and indirect impact quantified here represents a best case or maximum impact. Further work based on intensity and force of infection based on the work of French et al [16] is required to refine and triangulate the estimate. Estimating only the direct effects of treatment in the treated population provides a conservative estimate of infection averted. Due to the definition of prevalence used in the study and the data available, the prevalence estimate in the treated population is under estimated (and consequently cost per treatment is overestimated). The true value of the infections averted is believed to be between these two estimates. There is a 40–42% difference in the cost per infection averted between the “best” and worst case scenarios. However the magnitude of difference underlines the importance of refining the method and developing more robust estimates.

The most effective means of delivering helminth treatment to school age children has been debated in various papers. The Partnership for Child Development (PCD) [6] provided evidence from Ghana and Tanzania on the cost of large scale treatment in schools and the potential savings in using the existing school infrastructure for treatment. For many recipients, access to the more numerous schools is more convenient than attending more distant health facilities [19]. However, where school enrolment is low and particular groups (for example girls or the poorest children) are under-represented, there is a need for additional methods of reaching target populations [7]. Studies in Tanzania [20] and Uganda [21] have examined the effectiveness of Community Directed Treatment (ComDT) and school based treatment in terms of coverage for enrolled and non enrolled children. In Tanzania coverage in both systems was similar, whilst in Uganda coverage rates under ComDT was higher; the associated costs are not discussed.

The evidence on the cost effectiveness of three recent large scale helminth MDA studies in Sub-Saharan Africa is summarised in Table 7. This presents the characteristics: treatment strategies, distribution methods, coverage levels, activities costed, study duration and the number of treatments rounds provided. Each of these affects the cost and technical effectiveness of the programme.

**Table 7:** Comparison of MDA costs of three vertical helminth control programmes in Sub Saharan Africa.

Background Parameters	Note	B. Faso	Uganda	Niger
Strategy	a	A	B	B
School net enrolment 2005	b	40%	n/a	42%
No. districts in costing paper		ALL	6	4
Treatments in study area & period		3,322,564	432,746	818,562
Study period (years)		2	3	2
Activities included in cost	c	1	1+	1,2,3,4,
National coverage	d	91%	79%	66%,78%
PPN treated in communities: schools		1.5∶1	0.6∶1	5.2∶1
PPN targeted in communities: schools	e	1.64∶1	0.85∶1	2.7∶1
Costs included	f	2, 3a	2,3	1,2,3
Discounted analysis employed		No	Yes	Yes
**Programme costs/treatment**				
Economic	g	n/a	n/a	0.54 (0.58)
Financial or programme cost	g	0.32	n/a	0.44 (0.48)
Drug cost	h	0.22	0.22	0.28
**Economic cost/treatment by system**				
School based	g	n/a	0.54	0.74 (0.76)
Community based	g	n/a	n/a	0.44 (0.46)
**Financial cost/treatment by system**				
School based	g	0.31	0.39	0.45 (0.47)
Community based		0.33	n/a	0.39 (0.41)
**Sub district programme delivery cost treatment**				
Cost/person school based delivery	i	(0.08)	0.16	(0.09) 0.11
Cost/person CDD delivery	i	(0.11)	n/a	(0.03) 0.05

Notes.

a A. All SAC 1 treatment over 2 years, B All SAC in target areas & key adults C SAC 2 or more treatments.

b Rates as reported by UN ISCED level. Uganda rates are considered to be more the SSA average of 68%.

c 1. MDA 2. mapping 3. M&E 4. Prevalence surveys 5. Screening.

d B. Faso coverage over 2 years (2004–2005), Uganda: coverage in pilot phase (2003), Niger: coverage in pilot phase (4-2004/4-2005) and second phase (4-2005/4-2006).

e Based on first year results in Niger and Uganda.

f 1. International support costs, 2. Programme expenditure and costs, 3. Government contribution a) cash & b) recurrent in kind costs (e.g. staff salaries and vehicle usage).

g see activities included () including international cost.

h Drug usage estimated in Uganda. In Niger usage is based on district registers and is not stated in B Faso.

i district costs and () sub district costs. Costs exclude drugs, Uganda delivery cost is reduced by 5% to allow for central overheads.

n/a not available n/r not relevant.

Sources: [4],[5],[16].

Prevalence and mapping data facilitate treatment prioritisation of endemic areas. This reduces the numbers treated, easing pressure on constrained budgets, but allows the option, to treat targeted at risk adults. Niger and Uganda used a targeted approach. Burkino Faso undertook a blanket approach and treated all school children in the first treatment.

Burkina Faso has the lowest financial cost per treatment but excludes start up, mapping and M&E costs included in the studies (see Table7); it also has the highest coverage. Coverage is a key factor in determining costs per treatment; in particular capital and recurrent costs. Children accounted for all treatments in Burkina Faso and 53% of treatments in the Niger study. Niger's targeted strategy reduced the numbers of children requiring treatment. This eased the budget constraint allowing the targeted treatment of adults, and increased the scale economies of the programme.

Niger and Uganda provide a measure of the effectiveness of treatment. Infections averted are used based on anaemia in Uganda and schistosomiasis in Niger. The use of a technical measure of effectiveness provides the opportunity to assess, both ex-ante and ex-post the potential cost effectiveness of alternative strategies.

The sub-district school based delivery cost per treatment is similar between Niger and Burkina Faso (a difference of 10%). Uganda district school based delivery costs (allowing for central programme costs) are almost 45% greater than Niger's. The reason for this is not clear. It may be that central costs are included in activities other than “programme costs” Sub district community delivery cost per treatment in Niger are significantly lower than Burkina Faso due to the high numbers targeted and treated. The low levels of enrolment, low school numbers targeted and lower coverage rates all add to the relative cost per person of the Niger school based system.

The cost per person treated can be reduced either by increasing national coverage or by an improvement in resource efficiency. Alternative means of school based implementation are available and school based treatment can be delivered by teachers, health workers or CDDs, training may or not be required annually. However the differences in delivery will impact on treatment acceptability [19] and coverage, collaboration and motivation as well as the variable costs.

The distribution of programme costs in Niger, suggest cost savings would have greatest impact in drugs and training. Drugs are a major component of the treatment cost and account for almost half of the economic cost in the current study, between 27%–46% per district of the economic cost in Uganda and 69% of the financial cost in Burkina Faso. The central procurement (undertaken in 2004/06 by SCI) improved the buyer's market power, described by Fenwick and Thompson [12]. On average 3.1 praziquantel tablets and 1.4 albendazole tablets were consumed per treatment in the current study. Using an estimate of actual against planned praziquantel usage (3.5 tablets/adult and 2.5 tablets/child), gave an average difference of 6% with a range of −4% to 23% across the four districts. The average rate is considerably more than the wastage rate of 1% assumed in [5]. This range shows a considerable discrepancy between districts and emphasises the importance of robust drug monitoring and reporting system.

Targeted treatment of at risk adults in high endemic areas is used in Niger and Uganda in line with WHO guidelines. As the cost per infection averted in adults was 3.5 greater than for a child, it is important to understand the economic value of adult treatment. One approach is to assess the direct benefits in terms of impact on adult productivity and the value of this productivity. USAID Famine Early Warning System Network (FEWS-NET): Niger Livelihoods Profiles, provide a useful description of livelihood profiles in these areas. Two studies, with agricultural production comparable to that in the Niger study, report the impact of schistosomiasis on agricultural labour productivity [22], [23]. The impact of schistosomiasis treatment on family labour in paddy rice growing systems in Mali [22] found that health is improved due to schistosomiasis treatment. As a result the time available for farm work by family workers increased by 69 days/ha. Much of this time was invested in the cultivation of additional non irrigated land, (0.47 ha) (if it is assumed a family has 7-10 members, the average improvement / person would be 10-7 days.). The days of family farm labour lost due to schistosomiasis and other parasitic and non parasitic infections was assessed in rain fed farm systems in Benue State, Nigeria [23]. In these systems 46% of time lost due to illness was related to schistosomiasis, an average loss of 18.7 working days per adult. The cost per adult of infection averted in Niger in the first round is estimated here as $4.3 (Table 6) equivalent to 3 days of labour (based on the agricultural day rate ($1.4) in 2005, a year of famine) or 2.3 days (based on rate of $1.9 in a normal year). This indicates the potential economic net gain from adult treatment. The gain could be greater depending on adult rates of re-infection and consequent treatment need.

### Conclusion

The cost of treatment per person is driven by the scale of treatment. The strategy, in Niger, to include targeted adults as well as school age children has increased the treatment numbers and reduced the cost per person treated and increased effectiveness. A conservative estimate of cost effectiveness over 2 years for the treated population is estimated to be $1.1 per infection averted for children and $6.5 for adults.

This study used a targeted treatment strategy; 53% of treatments were to children, but only 16% of the population treated received school based treatment. Under these conditions community based treatment was more cost effective than school based treatment. In Burkina Faso, only school age children were treated; 40% of these received school based treatment); the school based system was more cost effective per treatment. However, the school and community based distribution systems serve overlapping groups in the population; and was designed to facilitate access to treatment for different groups and support a coverage rate of 75% or more in target populations. Any improvement in either system must be the result of improved resource use or increased coverage at the district and programme level if the change is not to impact on the effectiveness of the other system.

In designing cost effective and sustainable programmes factors relating to: the treatment strategy, the demographic mix of the population served, system acceptability to stakeholders and the coverage rate need to be taken into account along with logistic issues such as health staff availability. Economic and financial assessment of alternative implementation plans should be undertaken for the project or programme design. This would support decision makers and programme managers, provide financial evidence in planning discussions and negotiations and potentially reduce the need for programme changes to improve cost effectiveness.

## Supporting Information

Table S1 **Summary of principal programme unit costs.**
(RTF)

Table S2 **Life of capital assets.**
(RTF)

Table S3 **Mean prevalence and confidence limits of baseline and follow up surveys in study areas.** Table S3 provides further detail of the mean and 95% confidence intervals used to estimate the cases averted in estimating the costs per case averted. In Gaya, Dosso region the prevalence rates are higher in the second follow up compared with the first. The reason is not known, but may relate to difference in coverage in the surveyed school. Coverage is available at district level, and sub district data is collated at district level, but is difficult to obtain retrospectively. The followed up for children over the two years was high (81%).
(RTF)

Table S4 **Glossary of terms.**
(RTF)

## References

[1] Canning D (2006). Priority setting and the ‘neglected’ tropical diseases.. Transactions of the Royal Society of Tropical Medicine and Hygiene.

[2] Montresor A, Gabrielli AF, Diarra A, Engels D (2010). Estimation of the cost of large scale deworming programmes with benzimidazoles.. Transactions of the Royal Society of Tropical Medicine and Hygiene.

[3] Walker D, Fox-Rushby J (2000). Economic evaluation of parasitic diseases: A critique of the internal and external validity of published studies.. Tropical Medicine and International Health.

[4] Gabrielli AF, Touré S, Sellin B, Sellin E, Ky C (2006). A combined school- and community-based campaign targeting all school-age children of Burkina Faso against schistosomiasis and soil-transmitted helminthiasis: Performance, financial costs and implications for sustainability.. Acta Tropica.

[5] Brooker S, Kabatereine NB, Fleming F, Devlin N (2008). Cost and cost-effectiveness of nationwide school-based helminth control in Uganda: intra-country variation and effects of scaling-up.. Health Policy and Planning.

[6] The Partnership for Child Development (1999). The cost of large-scale school health programmes which deliver anthelmintics to children in Ghana and Tanzania.. Acta Tropica.

[7] Huseinl MH, Talaat M, El-Sayed MK, El-Badawi A, Evans DB (1996). Who misses out with school-based health programmes?. A study of schistosomiasis control in Egypt Transactions Of The Royal Society Of Tropical Medicine And Hygiene.

[8] Talaat M, Evans DB (2000). The costs and coverage of a strategy to control schistosomiasis morbidity in non-enrolled school-age children in Egypt.. Transactions of the Royal Society of Tropical Medicine and Hygiene.

[9] Guyatt H, Evans D, Lengeler C, Tanner M (1994). Controlling schistosomiasis: the cost-effectiveness of alternative delivery strategies.. Health Policy Plan.

[10] Fenwick A, Webster JP, Bosque-Oliva E, Blair L, Fleming F (2009). The Schistosomiasis Control Initiative (SCI): rationale, development and implementation from 2002–2008.. Parasitology.

[11] Garba A, Touré S, Dembelé R, Bosque-Oliva E, Fenwick A (2006). Implementation of national schistosomiasis control programmes in West Africa.. TRENDS in Parasitology.

[12] Frost LJ, Reich MR (2008). Access: How do good health technologies get to poor people in poor countries?. HCPDS, chapter.

[13] Jamison DT (2006). Disease Control Priorities in Developing Countries.

[14] Montresor A, Crompton DWT, Hall A, Bundy DAP, Savioli L (1998). Guidelines For The Evaluation Of Soil-Transmitted Helminthiasis And Schistosomiasis At Community Level..

[15] Touré S, Zhang Y, Bosqué-Oliva E, Ky C, Ouedraogo A (2008). Two-year impact of single praziquantel treatment on infection in the national control programme on schistosomiasis in Burkina Faso.. Bulletin of the World Health Organization.

[16] French MD, Churcher TS, Gambhir M, Fenwick A, Webster JP (2010). Observed Reductions in Schistosoma mansoni Transmission from Large-Scale Administration of Praziquantel in Uganda: A Mathematical Modelling Study.. PLoS Negl Trop Dis.

[17] Anderson RM, May RM (2005). Infectious Diseases of Human Dynamics and Control.

[18] Miguel E, Kremer M (2004). Worms: identifying impacts on education and health in the presence of treatment externalities.. Econometrica.

[19] Nwaorgu OC, Okeibnos J, Madu E, Amazingo U, Onyegegbu N (1998). School based schistosomiasis and intestinal helminthiasis control programme in Nigeria: acceptability to community members.. Tropical Medicine and International Health.

[20] Massa K, Olsen A, Sheshe A, Ntakamulenga R, Ndawi B (2009). Can coverage of schistosomiasis and soil transmitted helminthiasis control programmes targeting school-aged children be improved?. New approaches Parasitology.

[21] Ndyomugyenyi R, Kabatereine N (2003). Integrated community-directed treatment for the control of onchocerciasis, schistosomiasis and intestinal helminth infections in Uganda: advantages and disadvantages.. Tropical Medicine and International Health.

[22] Audibert M, Etard JF (1998). Impact of Schistosomiasis on Rice Output and Farm Inputs in Mali.. Journal Of African Economies.

[23] Umeh JC, Amali O, Umeh EU (2004). The socio-economic effects of tropical diseases in Nigeria.. Economics and Human Biology.

