# Correction: Schistosomiais and Soil-Transmitted Helminth Control in Niger: Cost Effectiveness of School Based and Community Distributed Mass Drug Administration

**DOI:** 10.1371/annotation/30823662-77ac-4ac3-bd32-6f300121ac3f

**Published:** 2012-04-19

**Authors:** Jacqueline Leslie, Amadou Garba, Elisa Bosque Oliva, Arouna Barkire, Amadou Aboubacar Tinni, Ali Djibo, Idrissa Mounkaila, Alan Fenwick

The word "Schistosomiasis" is misspelled in the article title. The correct title is: Schistosomiasis and Soil-Transmitted Helminth Control in Niger: Cost Effectiveness of School Based and Community Distributed Mass Drug Administration. The correct citation is: Leslie J, Garba A, Oliva EB, Barkire A, Tinni AA, et al. (2011) Schistosomiasis and Soil-Transmitted Helminth Control in Niger: Cost Effectiveness of School Based and Community Distributed Mass Drug Administration. PLoS Negl Trop Dis 5(10): e1326. doi:10.1371/journal.pntd.0001326

