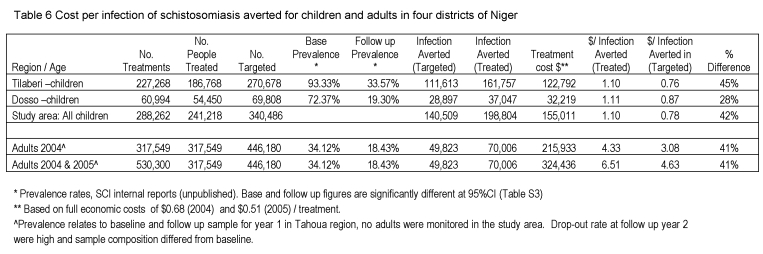# Correction: Schistosomiais and Soil-Transmitted Helminth Control in Niger: Cost Effectiveness of School Based and Community Distributed Mass Drug Administration

**DOI:** 10.1371/annotation/d9dce505-07d7-4d19-8c2f-9f707bddc287

**Published:** 2012-04-19

**Authors:** Jacqueline Leslie, Amadou Garba, Elisa Bosque Oliva, Arouna Barkire, Amadou Aboubacar Tinni, Ali Djibo, Idrissa Mounkaila, Alan Fenwick

There was a formatting error in Table 6. Please see the corrected Table 6 here: 

**Figure:**