# Supplementary material for: Schistosomiais and Soil-Transmitted Helminth Control in Niger: Cost Effectiveness of School Based and Community Distributed Mass Drug Administration
Source: PLoS Negl Trop Dis. 2011 Oct 11;5(10):e1326. doi: 10.1371/journal.pntd.0001326 (PMC3191121; doi:10.1371/journal.pntd.0001326)
Supplement: Table S1 — Summary of principal programme unit costs. (RTF) [file pntd.0001326.s001.rtf]

Category	Input 	Units 	Unit Cost $ 	
Minimum 	Maximum	
Capital Items 	Office building	Annualised cost  	      11,342 		
	Project vehicle	Purchase cost 	      24,669 	          48,251 	
	Computer 	Purchase cost 	        1,500 		
	Photo copier 	Purchase cost 	        5,104 		
Salaries 	 National  	
	Co-ordinator 	per month 	            417 		
	Central Technical Staff 	per month 	            125 	               330 	
	Central Administrative staff	per month 	            125 	               220 	
	Driver 	per month 	            110 		
	 District & Regional Health  	
	District & Regional Staff		            216 	               340 	
	Clinic Head Nurse 	per month 	            136 	               216 	
	Certified Nurse	per month 	              93 		
	Community Health Worker	per month 	              38 	                  66 	
	Distributer 	per month 	              38 	                  95 	
	 District & Regional Education 	
	Inspectorate Staff 	per month 	            244 	                309 	
	Teachers - civil servant 	per month 	            171 	                227 	
	Teacher -contracted 	per month 	              75 	                100 	
Allowances 	Central Technical Staff 	per day	              19 	                  28 	
	District Staff	per day	              19 		
	Training participant 	per session 	                5 		
	Community distributer 	per local distribution 	                5 		
	Driver 	per day	                5 		
Consumables 	Phone cards 	per card 	             1.9 		
	IEC poster	per unit 	             0.1 		
	Technical sheets 	per unit 	             0.1 		
	Treatment register 	per unit 	             0.2 		
	Dose poles 	per unit 	             2.8 		
	Radio broadcast	per broadcast 	           18.9 		
	Albendazole 	per dose *	           0.04		
	Praziquantel 	per dose *	           0.24		
	Hire rate / 4x4 (in district)	per day 	           70.9 		
	Hire rate / motor bike 	per day 	             9.5 		
	 Fuel 	per litre 	             1.1 		
* Average dose 1.4 tablets Albendazole, 3.1 tablets Praziquantel  includes adult & child consumption  and wastage 
