# Supplementary material for: Schistosomiais and Soil-Transmitted Helminth Control in Niger: Cost Effectiveness of School Based and Community Distributed Mass Drug Administration
Source: PLoS Negl Trop Dis. 2011 Oct 11;5(10):e1326. doi: 10.1371/journal.pntd.0001326 (PMC3191121; doi:10.1371/journal.pntd.0001326)
Supplement: Table S2 — Life of capital assets. (RTF) [file pntd.0001326.s002.rtf]

Asset  Category  	Years of life	
 Electrical and mechanical goods  	5 and 10	
 Furniture  	10	
 IT equipment	5	
 Medical equipment  short life  	5	
 Medical equipment  medium life  	10	
 Phone  	10	
 Vehicles  	5	
