# Supplementary material for: Schistosomiais and Soil-Transmitted Helminth Control in Niger: Cost Effectiveness of School Based and Community Distributed Mass Drug Administration
Source: PLoS Negl Trop Dis. 2011 Oct 11;5(10):e1326. doi: 10.1371/journal.pntd.0001326 (PMC3191121; doi:10.1371/journal.pntd.0001326)
Supplement: Table S3 — Mean prevalence and confidence limits of baseline and follow up surveys in study areas. Table S3 provides further detail of the mean and 95% confidence intervals used to estimate the cases averted in estimating the costs per case averted. In Gaya, Dosso region the prevalence rates are higher in the second follow up compared with the first. The reason is not known, but may relate to difference in coverage in the surveyed school. Coverage is available at district level, and sub district data is collated at district level, but is difficult to obtain retrospectively. The followed up for children over the two years was high (81%). (RTF) [file pntd.0001326.s003.rtf]

 Region/year                                             	95% Confidence Interval	
Tilaberi (7-15 yrs)	Mean	Lower	Upper		
Base line	93.33%	90.95%	95.72%		
Follow Up 1	45.48%	40.71%	50.24%		
Follow Up 2	33.57%	29.06%	38.09%		
Dosso (7-15 yrs)		
Base line	72.37%	66.56%	78.17%		
Follow Up 1	13.60%	9.15%	18.05%		
Follow Up 2	19.30%	14.18%	24.42%		
Adults 					
Base line	34.12 %	28.30%	39.94%		
Follow Up 1	18.43 %	13.67%	23.19%		
					
