# Supplementary material for: Schistosomiais and Soil-Transmitted Helminth Control in Niger: Cost Effectiveness of School Based and Community Distributed Mass Drug Administration
Source: PLoS Negl Trop Dis. 2011 Oct 11;5(10):e1326. doi: 10.1371/journal.pntd.0001326 (PMC3191121; doi:10.1371/journal.pntd.0001326)
Supplement: Table S4 — Glossary of terms. (RTF) [file pntd.0001326.s004.rtf]

Term 	Meaning	
Annualise	This is the cost per year of owning and operating an asset over its entire lifespan, sometimes called the equivalent annual cost	
Constant prices	A value from which the overall effect of general price inflation has been removed, also known as real prices. 	
Discounting	A mechanism by which values which occur in future years are weighted (set by the discount rate) to ensure that the time value of money is taken into account. 	
Discount rate	The economic opportunity cost of capital or the economic rate of return on alternative marginal projects or investments.	
Financial prices	These are the actual prices at which inputs are bought and outputs sold and are used in financial analysis. In economic analysis, where prices are distorted due to market or government failure, it is necessary to impute the price that reflects the real economic value of an input or output.  The opportunity cost may be used to reflect this value. 	
Net primary school enrolment (NER)	Enrolment of the official age group for a given level of education expressed as a percentage of the corresponding population.	
Opportunity cost:	The benefit or value that could have been gained from an alternative use of the same resource.	
Present value	Sum of one or more annual discounted values 	
